# Structural, Impedance and Electrochemical Characteristics of Electrical Double Layer Capacitor Devices Based on Chitosan: Dextran Biopolymer Blend Electrolytes

**DOI:** 10.3390/polym12061411

**Published:** 2020-06-24

**Authors:** Shujahadeen B. Aziz, Muhamad H. Hamsan, Muaffaq M. Nofal, Wrya O. Karim, Iver Brevik, Mohamad. A. Brza, Rebar T. Abdulwahid, Shakhawan Al-Zangana, Mohd F. Z. Kadir

**Affiliations:** 1 Advanced Polymeric Materials Research Lab., Department of Physics, College of Science, University of Sulaimani, Qlyasan Street, Sulaimani 46001, Kurdistan Regional Government, Iraq; mohamad.brza@gmail.com (M.A.B.); rebar.abdulwahid@univsul.edu.iq (R.T.A.); 2 Department of Civil Engineering, College of Engineering, Komar University of Science and Technology, Sulaimani 46001, Kurdistan Regional Government, Iraq; 3 Institute for Advanced Studies, University of Malaya, Kuala Lumpur 50603, Malaysia; hafizhamsan93@gmail.com; 4 Department of Mathematics and General Sciences, Prince Sultan University, P.O. Box 66833, Riyadh 11586, Saudi Arabia; muaffaqnofal@gmail.com; 5 Department of Chemistry, College of Education, University of Sulaimani, Old Campus, Sulaimani 46001, Kurdistan Regional Government, Iraq; wrya.karim@univsul.edu.iq; 6 Department of Energy and Process Engineering, Norwegian University of Science and Technology, N-7491 Trondheim, Norway; 7 Manufacturing and Materials Engineering Department, Faculty of Engineering, International Islamic University of Malaysia, Kuala Lumpur 50603, Gombak, Malaysia; 8 Department of Physics, College of Education, University of Sulaimani, Old Campus, Sulaimani 46001, Kurdistan Regional Government, Iraq; 9 Department of Physics, College of Education, University of Garmian, Kalar 46021, Iraq; shakhawan.al-zangana@garmian.edu.krd; 10 Centre for Foundation Studies in Science, University of Malaya, Kuala Lumpur 50603, Malaysia; mfzkadir@um.edu.my

**Keywords:** biopolymer blend electrolyte, XRD and FTIR study, impedance, EDLC study

## Abstract

This report presents the preparation and characterizations of solid biopolymer blend electrolyte films of chitosan as cationic polysaccharide and anionic dextran (CS: Dextran) doped with ammonium iodide (NH_4_I) to be utilized as electrolyte and electrode separator in electrical double-layer capacitor (EDLC) devices. FTIR and XRD techniques were used to study the structural behavior of the films. From the FTIR band analysis, shifting and broadening of the bands were observed with increasing salt concentration. The XRD analysis indicates amorphousness of the blended electrolyte samples whereby the peaks underwent broadening. The analysis of the impedance spectra emphasized that incorporation of 40 wt.% of NH_4_I salt into polymer electrolyte exhibited a relatively high conductivity (5.16 × 10^−3^ S/cm). The transference number measurement (TNM) confirmed that ion (t_ion_ = 0.928) is the main charge carriers in the conduction process. The linear sweep voltammetry (LSV) revealed the extent of durability of the relatively high conducting film which was 1.8 V. The mechanism of charge storage within the fabricated EDLC has been explained to be fully capacitive behavior with no redox peaks appearance in the cyclic voltammogram (CV). From this findings, four important parameters of the EDLC; specific capacitance, equivalent series resistance, energy density and power density were calculated as 67.5 F/g, 160 ohm, 7.59 Wh/kg and 520.8 W/kg, respectively.

## 1. Introduction

From the perspective of industrial technology, the miniaturization and flexibility of electrical appliances have been the subject of intense focus. For example, the increasing use of smartphones is considered to be one of the latest technologies worldwide. The heart of technology devices is the energy generator part; electrochemical double-layer capacitors (EDLCs) can be regarded as potential energy sources in many electronic devices. The superiority of EDLCs can be associated with a relatively long life-cycle, ease of fabrication, safety, and high performance [1,2]. Carbon materials have been used as the backbone materials in EDLC electrodes, activated carbon being the most popular one. Many attempts have been made to study the compatibility of this material with polymer electrolytes [3,4,5,6,7]. In addition, cost-effective, relatively high electronic conductivity and satisfactory chemical stability are among the unique characteristics of activated carbon [8]. Based on its large surface area, activated carbon provides a large double-layer as a result of ion accumulation at the interfacial region via an energy storage mechanism. This ion accumulation at the interface region progresses via the well-defined phenomenon known as non-Faradaic reaction where ion adsorption occurs. From a basic viewpoint, there is an attraction between ions and electrons from the electrolyte and electrodes, respectively [9,10]. In fact, the performance of energy storage devices can be highly affected by the electrode materials. This encourages the researchers to improve the properties of electrode materials and try various biodegradable materials including eggshell, manganese dioxide, and conducting polymers [11,12,13,14]. The efforts are essentially concentrated on increasing the surface area, rising conductivity, electrochemical stability and good mechanical and thermal properties of the electrode material.

Nowadays, people use smart devices in many fields worldwide, for instance in education, communication, navigation, and entertainment. As a consequence, the demand for replacing large gadgets has increased dramatically. Orlins et al. have provided deep insights into the problem of electronic waste production and suggested proper solutions for a safer environment via collection of e-wastes and properly discarding smartphone devices [15]. It has been found that replacing the present polymers in these devices with a biodegradable one could solve this problem to a large extent [16]. Dextrans, synthesized by culturing *Leuconostocmesenteroides*, is widely employed as a biopolymer in medical applications as drug carriers. From a chemical structural view, they consist of linear polymer backbones where the monomer units are connected mainly with 1,6-α-D-glucopyranosidic linkages [17]. Ion gel electrolytes have been considered promising candidates to be utilized in electrochemical device applications [18,19,20]. The favorable features of these electrolytes are their non-flammability, relatively high electrochemical stability, high ionic conductivity, and thermal robustness [21,22]. The mechanical integrity of ionic gel liquids lacks the capability of encompassing low molecular weight gelators or more often macromolecules, forming ion gels [23]. Ionic conductivity and mechanical robustness are two key criteria for evaluating electrolytes. To allow low-voltage operation and fast response of electrochemical devices, high ionic conductivity has to be achieved. These properties can largely be manipulated by decisive selection of the polymer gelators [19,20,21,22,23,24]. These have been known to possess excellent film-forming capability because of enrichment in many oxygen functional groups in the dextran structure. In terms of the aforementioned properties, dextrans can be tuned to prepare a desired polymer electrolyte [25]. Hamsan et al. [26] have investigated a system of dextran-ammonium nitrate with conductivity up to 10^−5^ S/cm. 

Chitosan blended with additives is a widely used polymer, due to properties it shares with dextran, for example, non-toxicity, biodegradability, and relatively excellent film-forming ability. It is worth mentioning that crabs and prawns are the two main sources for obtaining this interesting polymer from the ocean [27].

Polymer blending is a promising and practical methodology that is commonly used for preparing polymeric materials with desirable properties which are impossible to achieve with individual polymer materials. The main reason for implementing this methodology is to enhance the mechanical strength as a consequence of structural modifications within the polymers at a molecular level [28]. Moreover, doping salts into polymer matrices results in a conduction process through free ions and amorphous region abundance. Shukur [29], proved that chitosan use in starch films decreases their amorphousness. It was well reported that lowering the degree of crystallinity of the polymer chain result in a substantial increase in the conductivity of an electrolyte film. The obvious difference between individual and blended polymers is that in the latter, sites that enable complexation with ions are available. It is well known that polymer blends are characterized by enrichment with many functional groups, showing high performance in numerous applications, such as tissue engineering, drug delivery, polymer electrolytes, and energy devices [30]. Generally, there are two sources of providing proton ions; first, strong inorganic acids, for example, sulfuric acid (H_2_SO_4_) and phosphoric acid (H_3_PO_4_); secondly, ammonium salts, for instance ammonium iodide (NH_4_I). On the one hand, the inorganic acid group possesses a relatively high conductivity but causes chemical degradation in the systems that added [31]. On the other hand, ammonium salts provide also a relatively high ionic conductivity with benign behavior [32]. For example, ammonium iodide (NH_4_I) has a low lattice energy value (605.3 kJ/mol), meaning a high degree of ion dissociation [33]. The dopant salt can considerably impact the DC conductivity of the prepared system. For example, a conductivity of 1.38 × 10^−3^ S/cm was recorded for chitosan (CS) and potato starch (PS) doped with ammonium fluoride (NH_4_F) [5], whereas dextran doped with ammonium nitrate (NH_4_NO_3_) system had a conductivity of 3 × 10^−5^ S cm^−1^ [26] and 1.28 × 10^−4^ S cm^−1^ was achieved for dextran-chitosan blend doped with ammonium thiocyanate (NH_4_SCN) [34]. In previous studies [34,35], a relatively low degree of crystallinity was obtained when 60 wt.% chitosan was mixed with 40 wt.% dextran. 

The current study is aimed at the influence of addition of various concentrations of NH_4_I into the 60 wt.% chitosan and 40 wt.% dextran blend system. The optimum ratio is recorded. Ultimately, a relatively high conductive blended polymer electrolyte system is utilized in the EDLC fabrication.

## 2. Materials and Methods 

### 2.1. Sample Preparation

Relatively high molecular weight (310,000–375,000) chitosan (CS) and dextran powder with average molecular weight (35,000 to 45,000) were purchased as raw materials from Sigma-Aldrich (Missouri, MO, USA). In the preparation of the polymer blend system (CS: dextran), 60 wt.% of CS and 40 wt.% of dextran were dissolved in two separate containers of 50 mL of 1% acetic acid with the aid of magnetic stirrer for 90 min at room temperature. Afterwards, these two solutions were mixed and stirred for 3 h to obtain a homogeneous blending solution. Optimization of the molar ratio of each component in the composite (CS: Dextran:NH_4_I) was performed using different quantities of ammonium iodide (NH_4_I) ranging from 10 to 40 wt.% in 10 wt.% steps. A series of codes were used to designate the prepared polymer blend electrolytes, whereby CSDPE0, CSDPE1, CSDPE2, CSDPE3, and CSDPE4 correspond to CS: Dextran incorporated with 0, 10, 20, 30, and 40 wt.% of NH_4_I, respectively. Ultimately, casting of the polymer blend electrolytes was carried out in different Petri dishes and they were left to evaporate the solvent at room temperature. To make sure the dryness of the films, a desiccator was used for, thereby producing solvent-free films.

### 2.2. Structural and Impedance Characterizations

**Figure 1:**
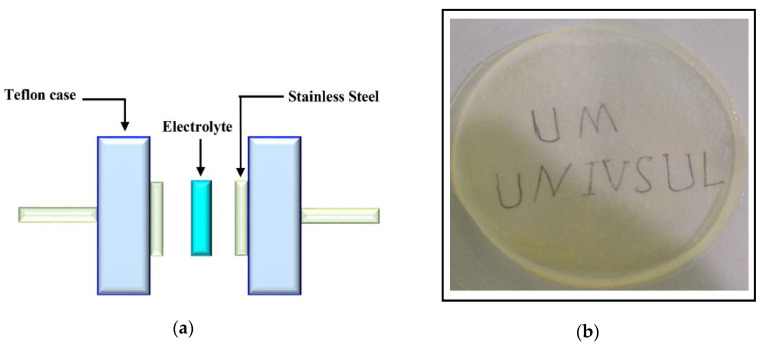
(**a**) Schematic diagram for impedance, TNM and LSV measurements. (**b**) Real image of a prepared SPE sample.

For the surface structure study of the polymer blend films, a D5000 X-ray diffractometer (1.5406 Å) was used (Bruker AXS GmbH, Berlin, Germany). The scanning runs of the samples were performed in the range of 10° to 80° (resolution = 0.1°) at 2θ angle. The confirmation of blend composite formation was conducted using a Spotlight 400 spectrometer (Perkin-Elmer, Waltham, MA, USA) in which the Fourier transform infrared (FTIR) spectra in the range of 450–4000 cm^−1^ with a resolution of 1 cm^−1^ were obtained. In the electrochemical properties study of the samples, the electrochemical impedance spectroscopy (EIS) was used by acquiring the spectra using a 3532–50 LCR HiTESTER (Hioki, Nagano, Japan) from 50 Hz to 5 MHz. The cell arrangement for EIS is shown in Figure 1a and a real SPE sample image is presented below in Figure 1b. The thicknesses of the samples ranged from 151–153 μm.

### 2.3. Transference Number Measurement (TNM)

Determination of ion (*t_ion_*) and electron (*t_el_*) transference numbers were carried out from cell polarization consisting of a stainless steel (SS)|high conducting SPE|SS by holding the voltage at 0.20 V. For this purpose, a DP3003 digital DC power supply (V&A Instrument, Shanghai, China) was used and the *t_ion_* was calculated at room temperature using the following equations [35]:

(1) $$t_{ion}=\frac{I_{i}-I_{ss}}{I_{i}}$$

(2) $$t_{el}=1-t_{ion}$$

where: current at initial and steady state measured in (µA) are denoted as *I_i_* and *I_ss_*, respectively, in the TNM plot.

### 2.4. Linear Sweep Voltammetry (LSV) Study

To determine the electrochemical stability (potential window) of the SPE, linear sweep voltammetry (LSV) was recorded using SS|high conducting SPE|SS arrangement. The potential scan was run using a Digi-IVY DY2300 potentiostat (Neware, Shenzhen, China) at sweep rate of 10 mV/s at room temperature.

### 2.5. EDLC Fabrication

The first step in the electrode fabrication was drying in a mixing process. Activated carbon (3.25 g) and carbon black (0.25 g), respectively, were dried and mixed using planetary ball miller prior to pouring into a mixed solution of polyvinylidene fluoride (PVdF, 0.5 g) and N-methyl pyrrolidone (NMP, 15 mL). Subsequently, the mixture was stirred vigorously for a few hours until a thick black solution was obtained. Then, the solution was used for coating aluminum foil (current collector) using a doctor blade and followed by drying in an oven at 60 °C. For further dryness, the electrodes were left in a desiccator. A specific geometry of 2.01 cm^2^for the electrodes was chosen and again left in the desiccator containing silica gel. The general arrangement of the EDLC cell was AC electrode| high conducting SPE|AC electrode.

**Figure 2:**
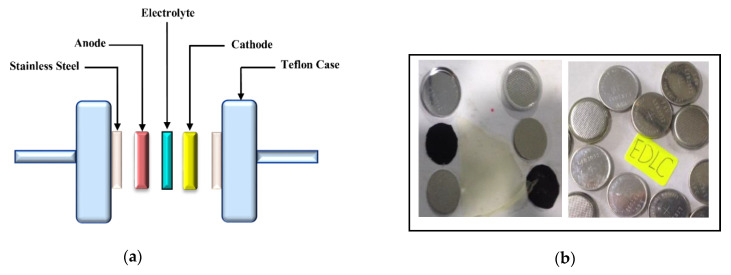
(**a**) Schematic illustration of the setup for CV and EDLC measurements. (**b**) Real images of the prepared EDLC systems.

The above arrangement was adjusted in packed form in a CR2032 coin cell. In preliminary measurements, cyclic voltammetry (CV) was recorded for the system. The schematic diagram of the EDLC cell and the realistic images of the fabricated EDLC systems are presented in Figure 2a,b, respectively. The conditions for CV andEDLCcharge-discharge examinations are a temperature of nearly 25 °C and relative humidity of nearly ~50%.

For the CV recording at the sweep rate of 100 mV/s, the Digi-IVY DY2300 potentiostat was used. The charge-discharge measurements were carried out as the second test using Neware battery cycler by applying the current density of 0.5 mA/cm^2^. Ultimately, four crucial parameters for EDLC evaluation were determined, such as specific capacitance (*C_s_*) in (F/g), equivalent series resistance (*ESR*) in (ohm), energy (*E*) in (Wh/kg) and power density (*P*) in (W/kg). All these were calculated from the following equations [35]:

(3) $$C_{s}=\frac{i}{gm}$$

(4) $$ESR=\frac{V_{d}}{i}$$

(5) $$E=\frac{C_{s}V}{2}$$

(6) $$P=\frac{V^{2}}{4m\left(ESR\right)}$$

Here *i*, *g*, *m* are respectively the applied current (1 mA), the gradient of discharge part, and the active material mass. The *V_d_* is the voltage drop before the discharge process initiates, and *V* is the applied voltage (0.9 V).

## 3. Results

### 3.1. XRD Study 

**Figure 3:**
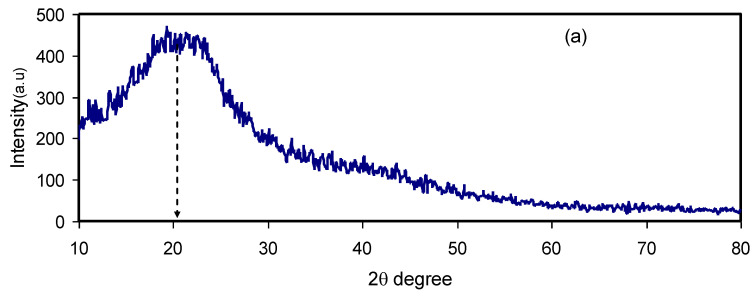

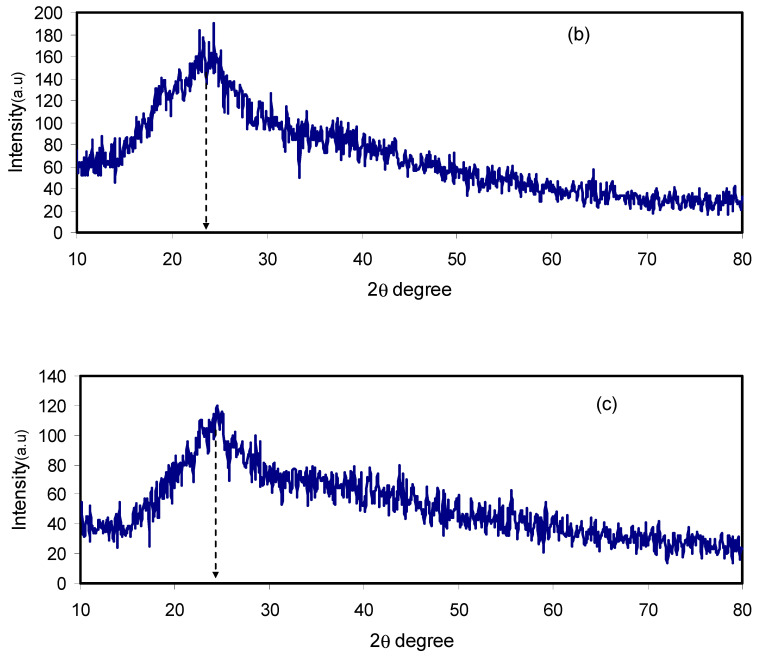
XRD pattern for (**a**) CSDPE0, (**b**) CSDPE2 and (**c**) CSDPE4 samples.

XRD patterns were recorded for structural analysis of the whole samples of pure CS and CS: Dextran films are shown in Figure 3a,b. It is evident that pure chitosan is characterized by a semi-crystalline structure. In the present study, the diffractogram of the pure chitosan sample exhibits a characteristic peak at 2θ = 21.0°, confirming the ordered structure. In other words, CS exists in a relatively high degree of crystallinity [36]. Figure 3a exhibits several numbers of crystalline peaks for the parent CS, which possesses two deep peaks centered at 2θ° = 15° and 20°, emphasizing the existence of a crystalline part in the parent chitosan membrane with an average intermolecular distance [37,38]. Chitosan rigidity comes primarily from its crystalline structure where there is hydrogen bonding (both intermolecular and intramolecular). The hydrogen bonding is formed between amino and hydroxyl groups as is inferred from absorbed water molecules [39,40].

From the XRD patterns of dextran it can be seen that a broad peak appears at 2θ = 18^o^ that corresponds to an amorphous region [41,42]. On the other hand, for CS: dextran blend composites, the XRD patterns exhibit only one concave peak and a few small peaks, as shown in Figure 3b. The broad hollows of CS: Dextran blend composites exhibit complete amorphous structure formation [43,44]. A more interesting observation is the shifting of the peak, indicating a phase transformation within the chitosan structure after blending [45].

The XRD patterns for chosen blend electrolytes are shown in Figure 3b,c. There is a substantial lowering in peak intensity with the incorporation of 20 wt.% of inorganic NH_4_I salt into CS: Dextran matrix as presented inFigure 3b. This is convincing evidence of an increased amorphous region within the polymer blend [46]. Moreover, the conductivity can be increased via increasing the amorphous region within polymer electrolytes. In other words, rapid segmental motions of the polymeric chain occur in the amorphous region. Based on this, charge carrier mobility increases which in turn results in high ionic conduction [47]. Figure 3c shows the impact of incorporation of the 40 wt.% of NH_4_I salt into the polymer electrolyte whereas a broader hollow exists. As a consequence, both peak broadening and intensity dropping are satisfactory evidence to confirm an amorphous region enhancement within the polymer system. To evaluate the trend of conductivity of the electrolytes, XRD pattern provides convinced evidence [40].

To lower the degree of crystallinity, it is possible to add inorganic salts into the CS: Dextran blends. This could be due to the disruption of the electrostatic interaction between the polar groups within the polymer matrices in one side and the cations of the salts on the other side. This means that relatively loose interactions exist within the polymer matrices as the hydrogen-bonding is weakened [48]. As a consequence, the degree of crystallinity is evident from the feature of the peaks. Hodge et al. have stated that two features; peak intensity and peak broadening, can be used in recognizing the extent of crystallinity within polymers [49]. In the current work the impact of NH_4_I salt on the crystallinity of the polymer membranes has been explained from the XRD pattern analysis.

### 3.2. FTIR Analysis

**Figure 4:**
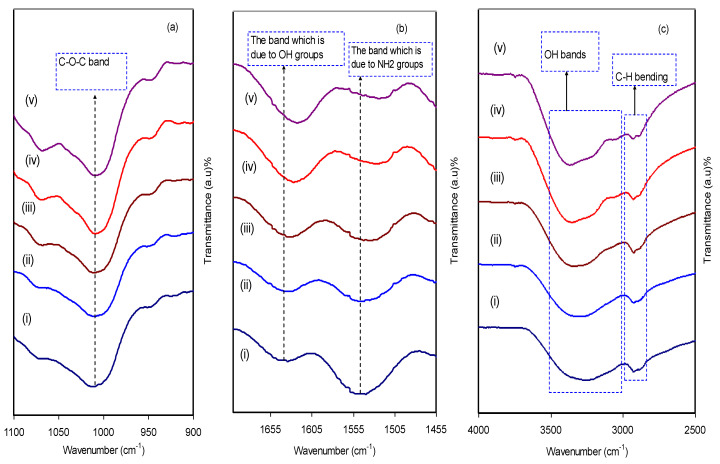
Spectra of FTIR for (i) CSDPE0, (ii) CSDPE1, (iii) CSDPE2, (iv) CSDPE3, and (v) CSDPE4 in the range (**a**) 900 to 1100 cm^−^^1^, (**b**) 1455 to 1700 cm^−^^1^, and (**c**) 2500 to 4000 cm^−^^1^.

The extent of the interactions between dopant and polymer matrix can be proved via Fourier transform infrared (FTIR) spectroscopy. The technique is based on interpreting the stretching or bending vibration modes of a wide range of bonds that are responsible for the signals in a spectrum. FTIR is a powerful and straightforward technique by which one can gain insight into strength of intermolecular interactions. Figure 4a–c show FTIR spectra of the pure CS: Dextran and blended electrolyte films in three different regions. The interesting band peak at 1086 cm^−^^1^ is a main characteristic feature of both vibrations of C–O–C bond in the glycosidic bridge, as exhibited in Figure 4a [50]. Convincing evidence of the existence of OH groups is the appearance of a broad band at 3351cm^−^^1^ as shown in Figure 4c [51,52]. Figure 4 calso exhibits a band at 2906 cm^−^^1^that is related to C–H stretching [51,52,53]. In Figure 4b, there are two other peaks that appear at 1651 and 1554 cm^−^^1^ which correspond to carboxamide (O=C–NHR) and amine (NH_2_) bands, respectively [54]. The peak at 1550 cm^−^^1^ is associated to N–H bending of a primary amide (−NH_2_) [36]. It is interesting to note that as the quantity of NH_4_I salt is increased, a number of peaks become broader their intensities are reduced as well. As an evidence, peak position shifting of the carboxamide (O=C–NHR) and amine (NH_2_) bands indicates a strong complexation between CS: Dextran and the dopant salt. 

In fact, the attachment of the cation to nitrogen and oxygen atoms is correlated to this lowering in the intensity of the vibration peak of the N–H or O=C–NHR bonds. Another confirmation of complexation between the polymer matrix and NH_4_I salt is the higher molecular weight of the functional group due to cation attachments in addition to peak position shifting and lowering in intensity as well [55].

Figure 4b shows the occurrence of both peak broadening and shifting to the lower wavenumber side which correspond to the carboxamide (O=C–NHR) and the amine (NH_2_) bands. Earlier studies have confirmed the strength of ion dissociation and thus high ionic conductivity. This leads to shift of bands to a lower wave number in the FTIR spectra [56,57,58]. Of incredible interest is ordering of the macromolecular that governs the intensity variation of these bands. Accordingly, these responses in the spectra, i.e., bands appearance in the spectra, of the complexes could result from the structural ordering [59].

### 3.3. Impedance Study

**Figure 5:**
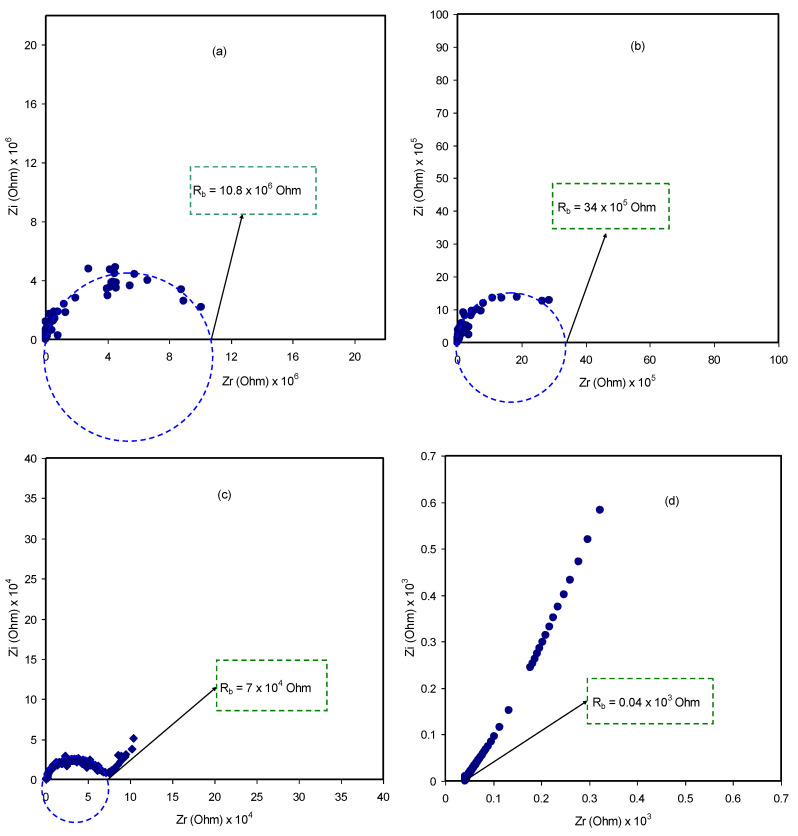
Impedance plots of (**a**) CSDPE1, (**b**) CSDPE2, (**c**) CSDPE3, and (**d**) CSDPE4 blended films at ambient temperature.

To analyse the electrochemical properties, especially the interfacial region of the system under study, electrochemical impedance spectroscopy (EIS) is a simple and suitable technique [60,61]. The impedance spectra of the blend electrolyte samples at ambient temperature are displayed in Figure 5a–d.

In the high frequency region, a semicircle appears and is modeled in the form of a parallel combination of both resistance (*R*_E_) and capacitance (*E*_C_) of the bulk electrolyte. The resistance and the capacitance responses are resulted from the migration process of proton ions and immobilized state of polymer chains, respectively.

**Figure 6:**
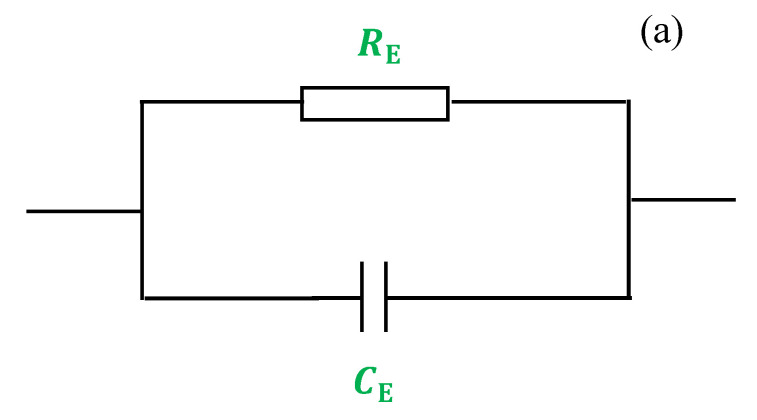

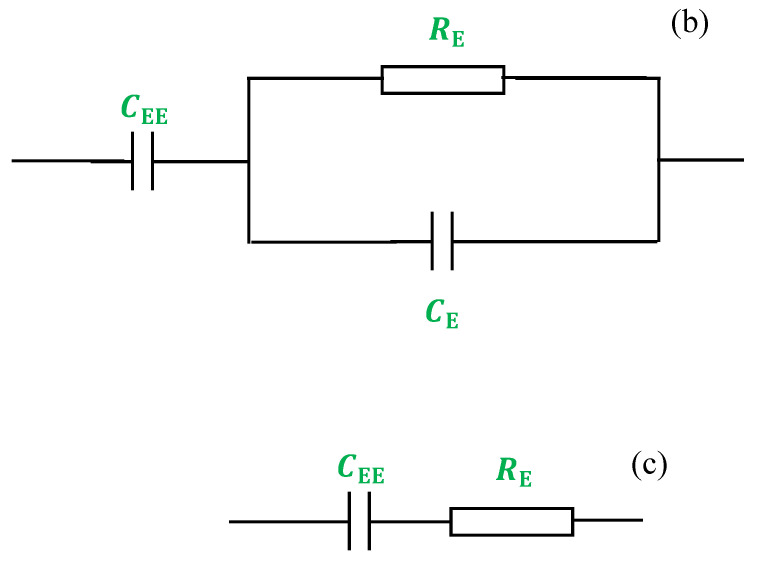
Schematic illustration of the electrical equivalent circuits (EECs) for (**a**) parallel combination of a resistor and capacitor, (**b**) parallel combination of a resistor and capacitor and in series with another capacitor and (**c**) series combination of a resistor and capacitor. Resistor is represented by the symbol 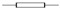 and capacitor represented by 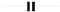.

A model of the whole electrical equivalent circuit (EEC) of blend electrolytes is presented in Figure 5a,b. The interesting point is that the diameter of the semicircle decreased with increasing quantity of NH_4_I salt and a tail appears at 30 wt.%. Accordingly, indication of the relaxation of ions with time distribution is proved from this semicircle size reduction and inclined spikes (see Figure 5c) [62]. In principle, the membrane electrolytes hold ion carriers from which the diffusion process through the membrane is facilitated when an AC electric field in a certain frequency range is applied. Ultimately, the tail results from ion accumulation building up at the interface of electrode/electrolyte region. Apparently, stainless steels are metallic electrodes within which a very large number of electrons can move freely and ions are not allowed to move across such kind of electrodes. All these phenomena cause the responses in the real and imaginary parts of the impedance patterns at various frequencies range [63]. Meanwhile, the ion accumulation at the interface region forms double-layer at this region where the electrode/electrolyte-like capacitor (*C*_EE_) results. Modeling these responses comprises a capacitor in series with the parallel combination of a resistor and another capacitor corresponding to the high frequency semicircle. The prediction of EEC for Figure 5c is seen schematically in Figure 6b.

Figure 5d shows the effect of 40 wt.% of NH_4_I, which is a relatively high salt concentration, on EIS response where the semicircle completely disappears. It is also proven that the polymer host body is responsible for resistive component [64]. The electrical profile of the system can be represented by two components; the resistor in series with electrode/electrolyte-like capacitor (*C*_EE_). The EEC of Figure 5d is presented in Figure 6c.

An increase of salt concentration lowers the bulk resistance significantly as exhibited in Figure 5a–d. For example, addition of NH_4_I up to 40 wt.% results in increased conductivity because of the increasing mobile charge carrier number. The salt addition increases the amorphous region within the polymer electrolyte. Clearly, it facilitates the ion transport and lowers the energy barrier as a consequence of the increase in amorphous region within the polymer electrolyte [65]. At the low frequency region, a spike shape feature in the EIS spectra indicates the diffusion process [66]. Apparently, as the salt concentration is increased, the bulk resistance lowers (see the insets in Figure 5. To calculate the *R_b_* values from the spectra, the point of intersection of semicircle with the real axis (Zr) is determined. Then, the sample DC conductivity (*σ_dc_*) based on the *R***_b_** value can be calculated. In the analysis of data point, the *R_b_* value and the sample dimensions have been used in the calculation of *σ_dc_* using the equation shown below [40]:

(7) $$\sigma_{dc}=\left(\frac{1}{R_{b}}\right)\times \left(\frac{t}{A}\right)$$

Here *t* and *A* represent the polymer electrolyte film thickness and the film surface area, respectively. The DC conductivity values of all the samples are presented in Table 1. The DC conductivity of the blend electrolyte is a decisive critical factor to be used in EDLCs. It is well-known that conductivity of an electrolyte is governed by both the number and the mobility of ions as shown in following equation [43,48,67]:

(8) σ=∑ηqμ

Here *ƞ*, *q* and *μ* are the carrier density in (cm^−^^3^), elementary charge in Coulomb and mobility in cm^2^/(V⋅s), respectively. From earlier work, it has been proven that the ammonium ion in polymer—ammonium salt system provides charge carrying species (H^+^ ion) [64]. The mechanism of proton conduction is described by Grotthuss. According to this theory, proton moves through ion exchanging between the complexed sites [68]. In other words, this mechanism states that a proton jumps over the complexing sites producing a vacant site followed by reorientation to occupy the vacant site [64]. It is clear that thenano-composite solid polymer electrolytes with low activation energy can provide a relatively easy ion migration through polymer electrolyte. Singh et al. prepared a PEO/AgI polymer electrolyte incorporated with Al_2_O_3_ and SiO_2_nano-fillers and recorded a conductivity of 5.78 × 10^−^^6^ and 7.032 × 10^−^^6^ S cm^−^^1^, respectively [69].

### 3.4. EDLC Study

#### 3.4.1. TNM Study

It is clarified that both electrons and ions contribute to the conductivity of the polymer electrolyte under study. Transference number measurements (TNMs) were carried out to prove the impact of ionic mobility and diffusion coefficient to the conductivity behavior of the electrolytes as well as the determining the identity of conducting species in the electrolyte. In this measurement, the identity of the main charge carrier in the polymer electrolyte is determined. In the measurement of TNM the working voltage was held at 0.2 V.

**Figure 7:**
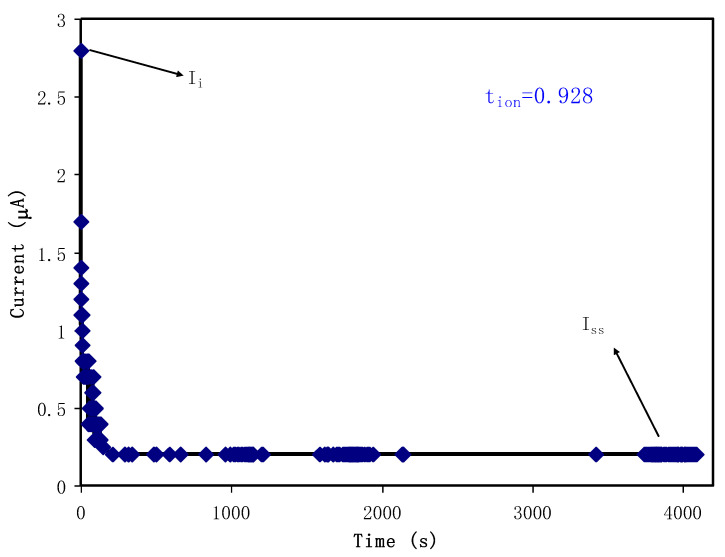
Polarization current versus time for the highest conducting CS: Dextran: NH_4_I electrolyte.

Figure 7 shows the polarization response of the relatively high conducting CS: Dextran: NH_4_I electrolyte film. From the polarization, it is noticed that a high current of 2.8 µA at the beginning is measured as result of the contribution of both ions and electrons. The TNM profile shows that the initial total current decreases as time progresses due to the diffusion layer depletion of the accumulated ionic species at the interface region and becomes constant in the fully depleted situation. This phenomenon is the characteristic of electrode response during electrochemical course that the ionic current through an ion-blocking electrode falls rapidly with time if the electrolyte is primarily ionic. Pinpointing this phenomenon as stated previously, the electrode used in this study was stainless steel (SS). Obviously, the current decays with time proceeding is resulting from the SS electrode blocking by ions where it becomes constant at 0.2 µA. This constant current for a period of time is known as steady-state current where there is a polarization of the electrodes [70]. To take advantage of this state, calculation of *t_ion_* and *t_el_* values is easy to perform using the aforementioned Equations (1) and (2). The values of *t_ion_* and *t_el_* are thus found to be 0.928 and 0.072, respectively. In electrochemistry, it is normal to have low *t_el_*, while *t_ion_* to be quite close to its ideal value of unity. Consequently, it is noted that ions are the main charge carriers in CS: Dextran: NH_4_I electrolyte, i.e., *t_ion_* > *t_el_*. This is essential to be utilized as electrolyte in EDLC, where ions accommodate at the interfacial region to form a doubl-layer, where in turn charge storing occurs. Vijaya et al. stated that as a benchmark, the ideal value of transference number for an electrolyte is in the range of 0.90 to 0.99, for example, for ammonium salt-based polymer electrolyte [71].

#### 3.4.2. LSV Study

**Figure 8:**
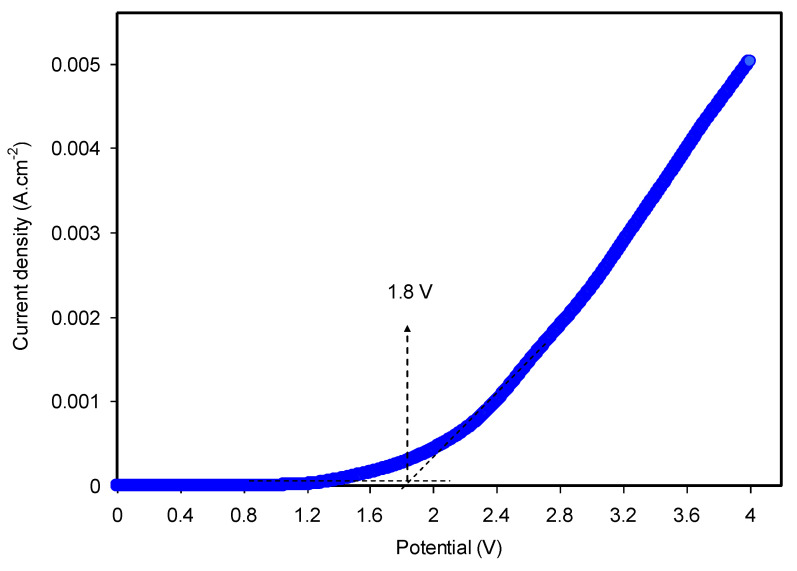
LSV curve for the highest conducting CS: Dextran: NH_4_I electrolyte at a scan rate 100 mV/s.

Determination of the potential window of the electrolyte under study is of significant importance for its utilization in energy devices. Each electrolyte has a characteristic potential window, i.e., there are two potential extremes which can be gained from LSV recording. The LSV response for the relatively high conducting CS: Dextran: NH_4_I electrolyte system at 10 mV/s is presented in Figure 8. It can be noted that the current reaches a constant value at low potential and then as the potential reaches 1.8 V, the current commences to rises sharply. This sharp rise of the current beyond 1.8 V is caused by the electrolyte decomposition. Fortunately, with this potential limit, it is satisfactory to use this polymer blend electrolyte in proton-based energy devices. It is worth-mentioning that the results obtained in the present study are comparable to other ammonium salt-based polymer electrolytes. Ng and Mohamad [72] also determined the decomposition potential for CS-based electrolyte, which was 1.8 V. In their study, ammonium nitrate (NH_4_NO_3_) and ethylene carbonate (EC) were used as the protonic electrolyte and plasticizer, respectively. In another study, Kadir and Arof [73], applied a CS: polyvinyl alcohol blend polymer electrolyte system in EDLC obtaining a potential stability up to 1.7 V. Hence, a satisfactory potential window for CS: Dextran: NH_4_I system was recorded which will be used in the fabrication of EDLCs.

#### 3.4.3. CV Study

**Figure 9:**
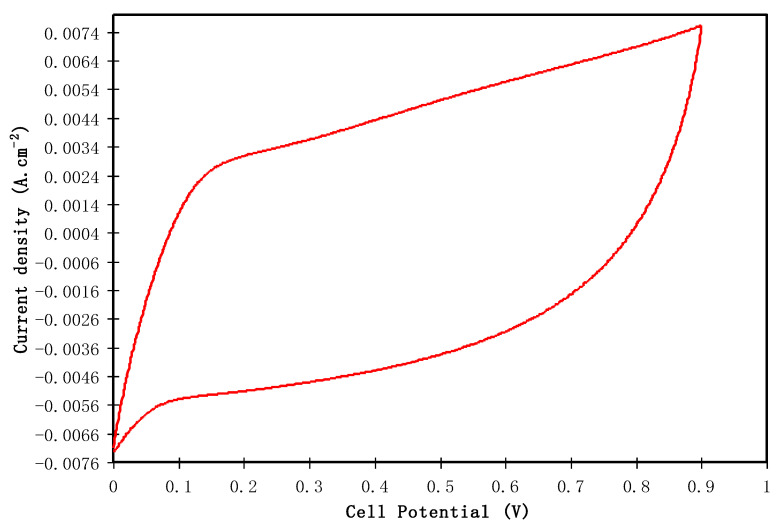
CV plot for the fabricated EDLC at 100 mV/s in the potential range of 0 to 0.9 V.

Cyclic voltammetry (CV) is frequently used as a powerful preliminary technique to characterize the capacitive behavior and differentiate between the non-Faradaic and Faradaic reactions [74]. Figure 9 shows the CV profile of the EDLC system in the potential window of 0 to 0.9 V at the scan rate of 100 mV/s. This CV profile of the fabricated EDLC system reflects an almost mirror image of the current responses to zero line, i.e., a symmetrical shape is obtained. The cause of this response is electric double-layer capacitive formation at the interfacial region, i.e., non-Faradaic behavior [75]. It is seen that there are no redox peaks over this potential range which indicates the non-existence of reduction and oxidation reactions in the system [76]. The CV profile is quite similar to the other EDLC systems where the CV shape is close to a leaf-like shape [77,78]. Yusof et al. [78] stated that the value of scan rate is a non-negligible factor that determines the shape of the CV profile. It is also worth mentioning that distorted perfect rectangular shape of the CV is determined by other factors, for instance electrode porosity and internal resistance [79]. Eftekhari stated that the charge accumulation mechanisms in supercapacitors (SCs) are complicated; hence, novel models have to be postulated and emphasized [80,81]. The problem of existing models for double layers from Helmholtz to Graham, is the impossibility of applying these theories on carbon-based capacitor systems [80]. This is because of the nature of the carbon electrodes that no possibility of double-layer charging in the micro-pores; other than the partial involvement of double-layer in greater pores or on graphene sheets [80].

#### 3.4.4. Charge-Discharge Study

**Figure 10:**
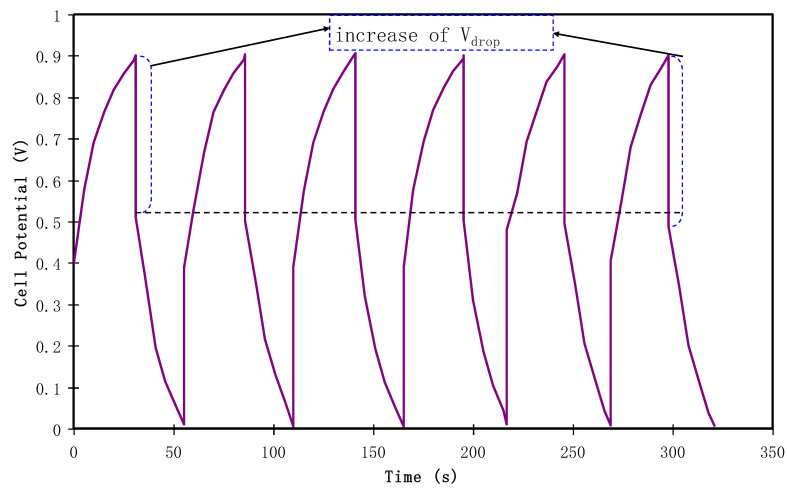
Charge-discharge profile for the fabricated EDLC at 0.5 mA/cm^2^.

**Figure 11:**
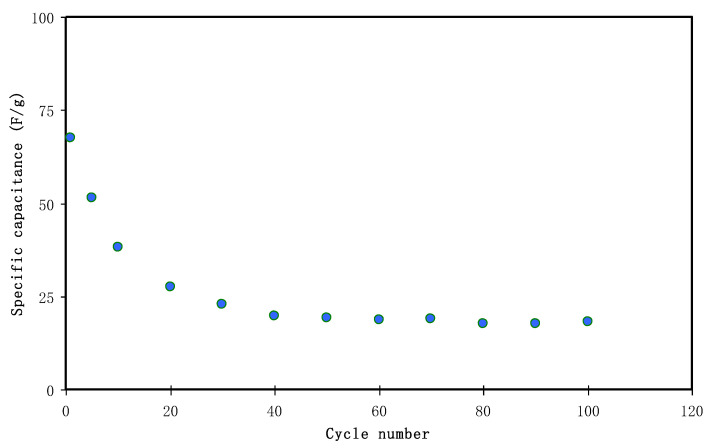
Specific capacitance of the assembled EDLC for 100 cycles.

The charge-discharge profile of the fabricated EDLC is shown in Figure 10. It is seen that the linear feature of the gradient of the curve indicates the existence of capacitance, resulting from an ionic double layer at the interfacial region between the active carbon electrodes and the electrolyte [82]. Importantly, it is also evident from the discharge curves that the linear characteristics of the assembled cell represent SCs [75]. Figure 11 shows the way of calculating specific capacitance (*C_s_*) of the EDLC using Equation (3). At the 1stcycle, *C_s_* = 67.5 F/g of the EDLC was obtained. As expected, by increasing the cycle number to 20, the *C_s_* value lowers to 27.4 F/g whereas beyond the 30th cycle, the value of *C_s_* starts to become nearly constant at 19.1 F/g. Liew and Ramesh have claimed that the overall performance of the EDLC is impacted by rapid charge-discharge process, including specific capacitance, energy density, and power density [83]. Presumably it is due to the lack of free ions as a consequence of ion pair formation or ion aggregation. Furthermore, under this condition, the ion adsorption process and potential energy storage at the electrode interface region have also been influenced. As a result, the electrical charge storage is based on the electrostatic interaction between electrons in the electrode side and ions in the electrolyte at the interfacial region which results in the formation of electric double-layer [30]. To figure out the system of interest, activated carbon was used in the fabrication of anode and cathode electrodes whereas the solid polymer films were used as the electrolyte and separator between the electrodes as well. This material possesses favorable properties, for example, cost-effective, relative high porosity, and desired surface area [75]. To be familiar with what have been reported in the literature, *C_s_* values of a number of the EDLCs are presented in Table 2. Herein, the results of CS: Dextran polymer blend showed more compatibility with NH_4_I compared to NH_4_F [35]. Additionally, suitability of the current electrolyte to be utilized in EDLC with relatively high specific capacitance is confirmed. The high specific capacitive value of the current system can be correlated to the low lattice energy of the salt. On the basis of low energy, one can expect high salt dissociation which is essential in this regard. To compare the lattice energy of two common salts, for example, NH_4_I and NH_4_F have 145.5 and 175.2 k Cal/mol, respectively, predicting strong attraction between NH_4_^+^ with F^−^ than I^−^ [84]. This reflects higher dissociation of NH_4_I than NH_4_F.

**Figure 12:**
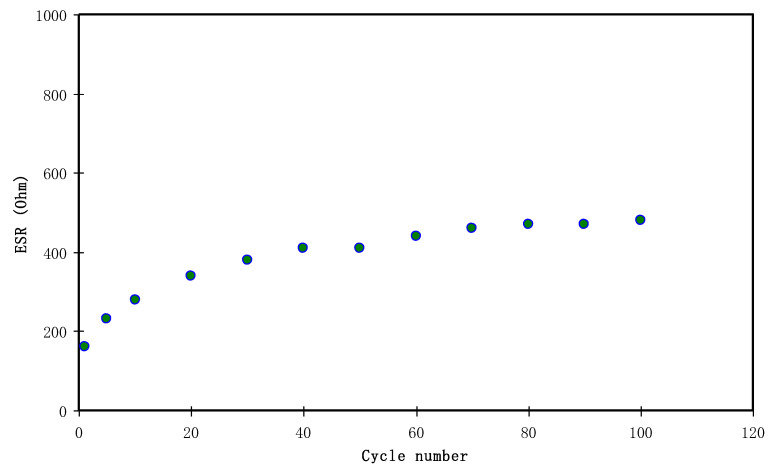
The pattern of equivalent series resistance of the EDLC for 100 cycles.

In Figure 12, a clear equivalent series resistance (ESR) is presented for the fabricated EDLC. From this finding, the EDLC cell exhibits a linear discharge behavior as well as small Ohmic drops, confirming its non-Faradic capacitive charge-storage mechanism [90,91,92]. From Equation (4), it is easy to calculate the ESR of the EDLC where the *V_d_* can be notified before discharging initiates as shown in Figure 10.

The internal resistance can be known using *ESR.* The value of *ESR* is governed by four parameters; first, the intrinsic resistance of the active electrode material, secondly the bulk resistance within the electrolyte, thirdly the current collector, and finally, the contact resistance between active material and electrolyte [93]. For the current study, a low value of 160 ohm for *ESR* was recorded. Lim et al. [94] documented an *ESR* of 199 Ohm for an EDLC based on polyvinyl alcohol-lithium perchlorate (LiClO_4_). The authors also claimed that a low *ESR* value implies a strong contact between the electrodes and electrolyte. Ajina and Isa [95] studied that the low value of *ESR* is caused by low percentage of PVDF insulator used in the electrode fabrication. It is worth noting that a high and constant value of *ESR* was recorded as to be at average value of 440 ohms as the cycle number exceeded 30thcycles while 340 ohm was recorded at 20th cycle. There is a consistency between *ESR* and specific capacitance as shown in Figure 11. Impressively, in this work; a constant value of ESR is still lower than that of NH_4_I-based EDLC. Yuhanees [96] documented *ESR* values from 2000 to 3000 ohm for EDLC with CS: starch: NH_4_I as the electrode separator. The author also claimed that the current leakage caused this increment in *ESR* at the side area of the EDLC which led to the electrolyte depletion.

**Figure 13:**
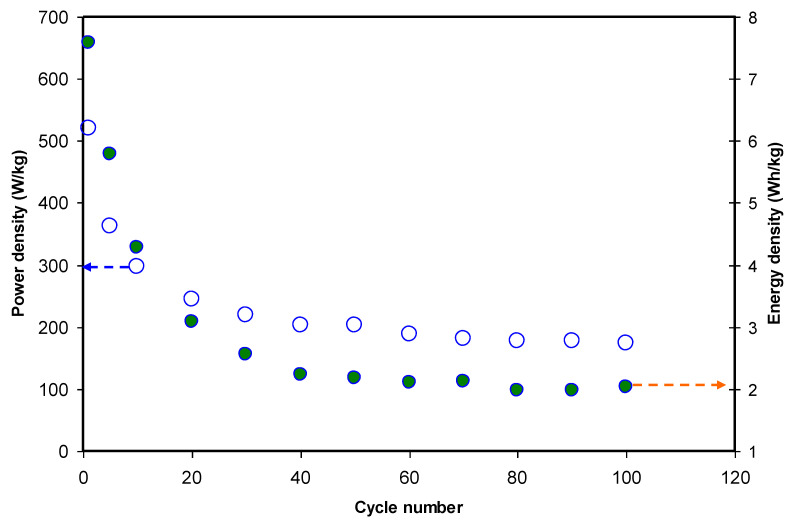
Energy density (E_d_) and Power density (P_d_) of the assembled EDLC up to 100th cycle.

Figure 13 presents the energy density (*E*) and power density (*P*) of the EDLC under study for 100 cycles. The E and P values were calculated using Equations (5) and (6). At the 1st cycle, the *E* value was 7.59 Wh/kg and it dropped to 5.8 and 3.1 Wh/kg at 5th and 20th cycles, respectively. It is familiar to us; the quantity of energy storage in EDLCs depends upon the ion accumulation at the interfacial region between the electrode surface and the electrolyte. The problem of ion accumulation is ascribed to less free ion availability as a consequence of growth of ion aggregates that substantially rise during fast charge-discharge process. Importantly, *E* is seen to be almost constant in the range of 2.0 to 2.2 Wh/kg beyond the 40th cycle. At this stage, ions have shown strong tendency to migrate towards the carbon electrodes with comparable energy barrier [97]. Aziz et al. [98], reported a constant value of *E* at 0.77 Wh/kg for a chitosan:MC:NH_4_I:glycerol-based EDLC.

Power density (*P*) is simply defined as the amount of energy that delivered per unit area. In Figure 13, *P* versus cycle number is exhibited. It is seen that at the 1 cycle, 520.8 W/kg has been recorded while 428.0 W/kg was documented for CS: Dextran: NH_4_F-based EDLC in a previous work [28]. Interestingly, *P* values were different for different dopant salts even the polymer blend host was the same. This could be ascribed to lower lattice energy of NH_4_I (637 kJ/mol) compared to NH_4_F (834 kJ/mol) [99,100]. The low lattice energy of the mentioned salt indicates its ease of dissociation; in other words, it provides a higher ion concentration for charge double layer accumulation at the interface, as a consequence, this results in higher EDLC performance. In this work, the rate of the EDLC power density has been observed to be constant at 186 W/kg and also there is a good agreement between the trend of *ESR* and the pattern of the EDLC parameters; *C_s_*, *E*, and *P*. It is seen that rise of internal resistance has been caused by the enactment of the EDLC.

## 4. Conclusions

In conclusion, it seems that biopolymer blend electrolytes of chitosan and dextran from *Leuconostocmesenteroides* are suitable mediators between electrodes in EDLC fabrication and produce high specific capacitance. A solution cast technique was employed for the construction of SPE films. The source of ions in the system was provided by ammonium iodide (NH_4_I). In the characterization, the Fourier-transform infrared spectroscopy (FTIR) and X-ray diffraction (XRD) techniques were used to confirm the complexation between the salt and the polymer blends. From the FTIR band analysis, shifting and broadening of the bands were observed with addition of increasing salt concentrations, proving complexation. The XRD analysis indicates the amorphousness of the blended electrolyte samples where the peaks underwent broadening. For studying the conductivity behavior of the membrane films, impedance spectroscopy was used. The salt incorporation into the polymer electrolyte increased the amorphousness and ionic conduction reached 5.16 × 10^−3^S/cm at 40 wt.% of NH_4_I. Based on the analysis of transference number measurements (TNMs), ion movement was found to be the main charge carriers in the conduction process, where the ionic transference number (*t_ion_*) was found to be around 0.928. The linear sweep voltammetry (LSV) technique showed the electrolyte was electrochemically stable up to 1.8 V. The mechanism of charge storage within the fabricated EDLC has been verified to be a fully capacitive behavior as no redox peaks appear in the CV plot. The trends of all EDLC crucial parameters were discovered to be consistent with the pattern of the equivalent series resistance and the EDLC under study can be qualified to be applicable at the large scale. The last finding is the influence of salt lattice energy on all parameters of the EDLC, such as specific capacitance, energy density, and power density. At the first cycle, 160 Ω was recorded for the ESR, and also a high value of 7.59 Wh/kg was obtained for energy density then stabilized at 3.1 Wh/kg. The power density was found to follow the same trend and finally stabilized at 186 W/kg up to 100 cycles.

## Figures and Tables

**Table 1:** Conductivity for pure CS: Ddextran and blended electrolyte films at room temperature.

Sample Designation	DCConductivity (S/cm)
CSDPE0	5.01 × 10^−^^10^
CSDPE1	2.73 × 10^−^^7^
CSDPE2	1.27 × 10^−^^5^
CSDPE3	5.62 × 10^−^^4^
CSDPE4	5.16 × 10^−^^3^

**Table 2:** Carbon-based EDLC studies.

Polymer Electrolyte	Specific Capacitance (F/g)	Reference
PVDF-ZnCl_2_	8.7	[85]
CS-Dex-NH_4_F	12.4	[35]
Nafion/PTFE composite polymer	16	[86]
CS-κcarrageenan-NH_4_NO_3_	18.5	[87]
Methycellulose-NH_4_NO_3_-PEG	25	[88]
CS-ιcarrageenan-H_3_PO_4_-PEG	30	[89]
CS-Dex-NH_4_I	67.5	This work
